# Community and Close Contact Exposures Associated with COVID-19 Among Symptomatic Adults ≥18 Years in 11 Outpatient Health Care Facilities — United States, July 2020

**DOI:** 10.15585/mmwr.mm6936a5

**Published:** 2020-09-11

**Authors:** Kiva A. Fisher, Mark W. Tenforde, Leora R. Feldstein, Christopher J. Lindsell, Nathan I. Shapiro, D. Clark Files, Kevin W. Gibbs, Heidi L. Erickson, Matthew E. Prekker, Jay S. Steingrub, Matthew C. Exline, Daniel J. Henning, Jennifer G. Wilson, Samuel M. Brown, Ithan D. Peltan, Todd W. Rice, David N. Hager, Adit A. Ginde, H. Keipp Talbot, Jonathan D. Casey, Carlos G. Grijalva, Brendan Flannery, Manish M. Patel, Wesley H. Self, IVY Network Investigators, CDC COVID-19 Response Team, IVY Network Investigators, Kimberly W. Hart, Robert McClellan, Hsi-nien Tan, Adrienne Baughman, CDC COVID-19 Response Team, Nora A. Hennesy, Brittany Grear, Michael Wu, Kristin Mlynarczyk, Luc Marzano, Zuwena Plata, Alexis Caplan, Samantha M. Olson, Constance E. Ogokeh, Emily R. Smith, Sara S. Kim, Eric P. Griggs, Bridget Richards, Sonya Robinson, Kaylee Kim, Ahmed M. Kassem, Courtney N. Sciarratta, Paula L. Marcet

**Affiliations:** ^1^CDC COVID-19 Response Team; ^2^Epidemic Intelligence Service, CDC; ^3^Influenza Vaccine Effectiveness in the Critically Ill (IVY) Network; ^4^Vanderbilt University Medical Center, Nashville, Tennessee; ^5^Beth Israel Deaconess Medical Center, Boston, Massachusetts; ^6^Wake Forest University Baptist Medical Center, Winston-Salem, North Carolina; ^7^Hennepin County Medical Center, Minneapolis, Minnesota; ^8^Baystate Medical Center, Springfield, Massachusetts; ^9^Ohio State University Wexner Medical Center, Columbus, Ohio; ^10^University of Washington Medical Center, Seattle, Washington; ^11^Stanford University Medical Center, Palo Alto, California; ^12^Intermountain Healthcare, Salt Lake City, Utah; ^13^Johns Hopkins Hospital, Baltimore, Maryland; ^14^University of Colorado School of Medicine, Aurora, Colorado.; Vanderbilt University Medical Center; Vanderbilt University Medical Center; Vanderbilt University Medical Center; Vanderbilt University Medical Center.; CDC COVID-19 Response Team; CDC COVID-19 Response Team; CDC COVID-19 Response Team; CDC COVID-19 Response Team; CDC COVID-19 Response Team; CDC COVID-19 Response Team; CDC COVID-19 Response Team; CDC COVID-19 Response Team; CDC COVID-19 Response Team; CDC COVID-19 Response Team; CDC COVID-19 Response Team; CDC COVID-19 Response Team; CDC COVID-19 Response Team; CDC COVID-19 Response Team; CDC COVID-19 Response Team; CDC COVID-19 Response Team; CDC COVID-19 Response Team; CDC COVID-19 Response Team.

Community and close contact exposures continue to drive the coronavirus disease 2019 (COVID-19) pandemic. CDC and other public health authorities recommend community mitigation strategies to reduce transmission of SARS-CoV-2, the virus that causes COVID-19 (*1*,*2*). Characterization of community exposures can be difficult to assess when widespread transmission is occurring, especially from asymptomatic persons within inherently interconnected communities. Potential exposures, such as close contact with a person with confirmed COVID-19, have primarily been assessed among COVID-19 cases, without a non-COVID-19 comparison group (*3*,*4*). To assess community and close contact exposures associated with COVID-19, exposures reported by case-patients (154) were compared with exposures reported by control-participants (160). Case-patients were symptomatic adults (persons aged ≥18 years) with SARS-CoV-2 infection confirmed by reverse transcription–polymerase chain reaction (RT-PCR) testing. Control-participants were symptomatic outpatient adults from the same health care facilities who had negative SARS-CoV-2 test results. Close contact with a person with known COVID-19 was more commonly reported among case-patients (42%) than among control-participants (14%). Case-patients were more likely to have reported dining at a restaurant (any area designated by the restaurant, including indoor, patio, and outdoor seating) in the 2 weeks preceding illness onset than were control-participants (adjusted odds ratio [aOR] = 2.4; 95% confidence interval [CI] = 1.5–3.8). Restricting the analysis to participants without known close contact with a person with confirmed COVID-19, case-patients were more likely to report dining at a restaurant (aOR = 2.8, 95% CI = 1.9–4.3) or going to a bar/coffee shop (aOR = 3.9, 95% CI = 1.5–10.1) than were control-participants. Exposures and activities where mask use and social distancing are difficult to maintain, including going to places that offer on-site eating or drinking, might be important risk factors for acquiring COVID-19. As communities reopen, efforts to reduce possible exposures at locations that offer on-site eating and drinking options should be considered to protect customers, employees, and communities.

This investigation included adults aged ≥18 years who received a first test for SARS-CoV-2 infection at an outpatient testing or health care center at one of 11 Influenza Vaccine Effectiveness in the Critically Ill (IVY) Network sites* during July 1–29, 2020 (*5*). A COVID-19 case was confirmed by RT-PCR testing for SARS-CoV-2 RNA from respiratory specimens. Assays varied among facilities. Each site generated lists of adults tested within the study period by laboratory result; adults with laboratory-confirmed COVID-19 were selected by random sampling as case-patients. For each case-patient, two adults with negative SARS-CoV-2 RT-PCR test results were randomly selected as control-participants and matched by age, sex, and study location. After randomization and matching, 615 potential case-patients and 1,212 control-participants were identified and contacted 14–23 days after the date they received SARS-CoV-2 testing. Screening questions were asked to identify eligible adults. Eligible adults for the study were symptomatic at the time of their first SARS-CoV-2 test.

CDC personnel administered structured interviews in English or five other languages^†^ by telephone and entered data into REDCap software (*6*). Among 802 adults contacted and who agreed to participate (295 case-patients and 507 control-participants), 332 reported symptoms at the time of initial SARS-CoV-2 testing and were enrolled in the study. Eighteen interviews were excluded because of nonresponse to the community exposure questions. The final analytic sample (314) included 154 case-patients (positive SARS-CoV-2 test results) and 160 control-participants (negative SARS-CoV-2 test results). Among nonparticipants, 470 were ineligible (i.e., were not symptomatic or had multiple tests), and 163 refused to participate. This activity was reviewed by CDC and participating sites and conducted consistent with applicable federal law and CDC policy.^§^

Data collected included demographic characteristics, information on underlying chronic medical conditions,^¶^ symptoms, convalescence (self-rated physical and mental health), close contact (within 6 feet for ≥15 minutes) with a person with known COVID-19, workplace exposures, mask-wearing behavior, and community activities ≤14 days before symptom onset. Participants were asked about wearing a mask and possible community exposure activities (e.g., gatherings with ≤10 or >10 persons in a home; shopping; dining at a restaurant; going to an office setting, salon, gym, bar/coffee shop, or church/religious gathering; or using public transportation) on a five-point Likert-type scale ranging from “never” to “more than once per day” or “always”; for analysis, community activity responses were dichotomized as never versus one or more times during the 14 days before illness onset. For each reported activity, participants were asked to quantify degree of adherence to recommendations such as wearing a face mask of any kind or social distancing among other persons at that location, with response options ranging from “none” to “almost all.” Descriptive and statistical analyses were performed to compare case-patients with control-participants, assessing differences in demographic characteristics, community exposures, and close contact. Although an effort was made initially to match case-patients to control-participants based on a 1:2 ratio, not all potential participants were eligible or completed an interview, and therefore an unmatched analysis was performed. Unconditional logistic regression models with generalized estimating equations with exchangeable correlation structure correcting standard error estimates for site-level clustering were used to assess differences in community exposures between case-patients and control-participants, adjusting for age, sex, race/ethnicity, and presence of one or more underlying chronic medical conditions. In each model, SARS-CoV-2 test result (i.e., positive or negative) was the outcome variable, and each community exposure activity was the predictor variable. The first model included the full analytic sample (314). A second model was restricted to participants who did not report close contact to a person with COVID-19 (89 case-patients and 136 control-participants). Statistical analyses were conducted using SAS software (version 9.4; SAS Institute).

Compared with case-patients, control-participants were more likely to be non-Hispanic White (p<0.01), have a college degree or higher (p<0.01), and report at least one underlying chronic medical condition (p = 0.01) (Table). In the 14 days before illness onset, 71% of case-patients and 74% of control-participants reported always using cloth face coverings or other mask types when in public. Close contact with one or more persons with known COVID-19 was reported by 42% of case-patients compared with 14% of control-participants (p<0.01), and most (51%) close contacts were family members.

**TABLE:** Characteristics of symptomatic adults ≥18 years who were outpatients in 11 academic health care facilities and who received positive and negative SARS-CoV-2 test results (N = 314)* — United States, July 1–29, 2020

Characteristic	No. (%)		P-value
	Case-patients (n = 154)	Control participants (n = 160)	
**Age group, yrs**			
18–29	44 (28.6)	39 (24.4)	0.18
30–44	46 (29.9)	62 (38.7)	
45–59	46 (29.9)	35 (21.9)	
≥60	18 (11.7)	24 (15.0)	
**Sex**			
Men	75 (48.7)	72 (45.0)	0.51
Women	79 (51.3)	88 (55.0)	
**Race/Ethnicity^†^**			
White, non-Hispanic	92 (59.7)	124 (77.5)	<0.01
Hispanic/Latino	29 (18.8)	12 (7.5)	
Black, non-Hispanic	27 (17.5)	19 (11.9)	
Other, non-Hispanic	6 (3.9)	5 (3.1)	
**Education (missing = 3)**			
Less than high school	16 (10.5)	3 (1.9)	<0.01
High school degree or some college	60 (39.2)	48 (30.4)	
College degree or more	77 (50.3)	107 (67.7)	
**At least one underlying chronic medical condition^§^**	75 (48.7)	98 (61.2)	0.01
**Community exposure 14 days before illness onset^¶^**			
Shopping	131 (85.6)	141 (88.1)	0.51
Home, ≤10 persons	79 (51.3)	84 (52.5)	0.83
Restaurant	63 (40.9)	44 (27.7)	0.01
Office setting	37 (24.0)	47 (29.6)	0.27
Salon	24 (15.6)	28 (17.6)	0.63
Home, >10 persons	21 (13.6)	24 (15.0)	0.73
Gym	12 (7.8)	10 (6.3)	0.60
Public transportation	8 (5.2)	10 (6.3)	0.68
Bar/Coffee shop	13 (8.5)	8 (5.0)	0.22
Church/Religious gathering	12 (7.8)	8 (5.0)	0.32
**Restaurant: others following recommendations such as wearing a face covering or mask of any kind or social distancing (n = 107)**			
None/A few	12 (19.0)	1 (2.3)	0.03
About half/Most	25 (39.7)	21 (47.7)	
Almost all	26 (41.3)	22 (50.0)	
**Bar: others following recommendations such as wearing a face covering or mask of any kind or social distancing (n = 21)**			
None/A few	4 (31.8)	2 (25.0)	0.01
About half/Most	7 (53.8)	0 (0.0)	
Almost all	2 (15.4)	6 (75.0)	
**Previous close contact with a person with known COVID-19 (missing = 1)**			
No	89 (57.8)	136 (85.5)	<0.01
Yes	65 (42.2)	23 (14.5)	
**Relationship to close contact with known COVID-19 (n = 88)**			
Family	33 (50.8)	5 (21.7)	<0.01
Friend	9 (13.8)	4 (17.4)	
Work colleague	11 (16.9)	6 (26.1)	
Other**	6 (9.2)	8 (34.8)	
Multiple	6 (9.2)	0 (0.0)	
**Reported use of cloth face covering or mask 14 days before illness onset (missing = 2)**			
Never	6 (3.9)	5 (3.1)	0.86
Rarely	6 (3.9)	6 (3.8)	
Sometimes	11 (7.2)	7 (4.4)	
Often	22 (14.4)	23 (14.5)	
Always	108 (70.6)	118 (74.2)	

* Respondents who completed the interview 14–23 days after their test date. Five participants had significant missingness for exposure questions and were removed from the analysis. Patients were randomly sampled from 11 academic health care systems that are part of the Influenza Vaccine Effectiveness in the Critically Ill Network sites (Baystate Medical Center, Springfield, Massachusetts; Beth Israel Deaconess Medical Center, Boston, Massachusetts; University of Colorado School of Medicine, Aurora, Colorado; Hennepin County Medical Center, Minneapolis, Minnesota; Intermountain Healthcare, Salt Lake City, Utah; Ohio State University Wexner Medical Center, Columbus, Ohio; Wake Forest University Baptist Medical Center, Winston-Salem, North Carolina; Vanderbilt University Medical Center, Nashville, Tennessee; John Hopkins Hospital, Baltimore, Maryland; Stanford University Medical Center, Palo Alto, California; University of Washington Medical Center, Seattle, Washington). Participating states include California, Colorado, Maryland, Massachusetts, Minnesota, North Carolina, Ohio, Tennessee, Utah, and Washington.

^†^ Other race includes responses of Native American/Alaska Native, Asian, Native Hawaiian/Other Pacific Islander, and other; these were combined because of small sample sizes.

^§^ Reported at least one of the following underlying chronic medical conditions: cardiac condition, hypertension, asthma, chronic obstructive pulmonary disease, immunodeficiency, psychiatric condition, diabetes, or obesity.

^¶^ Community exposure questions asked were “In the 14 days before feeling ill about how often did you:” with options of “shop for items (groceries, prescriptions, home goods, clothing, etc.)” (missing = 1); “have people visit you inside your home or go inside someone else's home where there were more than 10 people”; “have people visit you inside your home or go inside someone else's home where there were 10 people or less”; “go to church or a religious gathering/place of worship” (missing = 1); “go to a restaurant (dine-in, any area designated by the restaurant including patio seating)” (missing = 1); “go to a bar or coffee shop (indoors)” (missing = 2); “use public transportation (bus, subway, streetcar, train, etc.)” (missing = 1); “go to an office setting (other than for healthcare purposes)” (missing = 1); “go to a gym or fitness center” (missing = 1); and “go to a salon or barber (e.g., hair salon, nail salon, etc.)” (missing = 1). Response options were coded as never versus at least once in the 14 days prior to illness onset. Some participants had missing data for exposure questions.

** Other includes patients of health care workers (9), patron of a restaurant (1), spouse of employee (1), day care teacher (1), member of a religious congregation (1), and unspecified (1).

Approximately one half of all participants reported shopping and visiting others inside a home (in groups of ≤10 persons) on ≥1 day during the 14 days preceding symptom onset. No significant differences were observed in the bivariate analysis between case-patients and control-participants in shopping; gatherings with ≤10 persons in a home; going to an office setting; going to a salon; gatherings with >10 persons in a home; going to a gym; using public transportation; going to a bar/coffee shop; or attending church/religious gathering. However, case-patients were more likely to have reported dining at a restaurant (aOR = 2.4, 95% CI = 1.5–3.8) in the 2 weeks before illness onset than were control-participants (Figure). Further, when the analysis was restricted to the 225 participants who did not report recent close contact with a person with known COVID-19, case-patients were more likely than were control-participants to have reported dining at a restaurant (aOR = 2.8, 95% CI = 1.9–4.3) or going to a bar/coffee shop (aOR = 3.9, 95% CI = 1.5–10.1). Among 107 participants who reported dining at a restaurant and 21 participants who reported going to a bar/coffee shop, case-patients were less likely to report observing almost all patrons at the restaurant adhering to recommendations such as wearing a mask or social distancing (p = 0.03 and p = 0.01, respectively).

**FIGURE:**
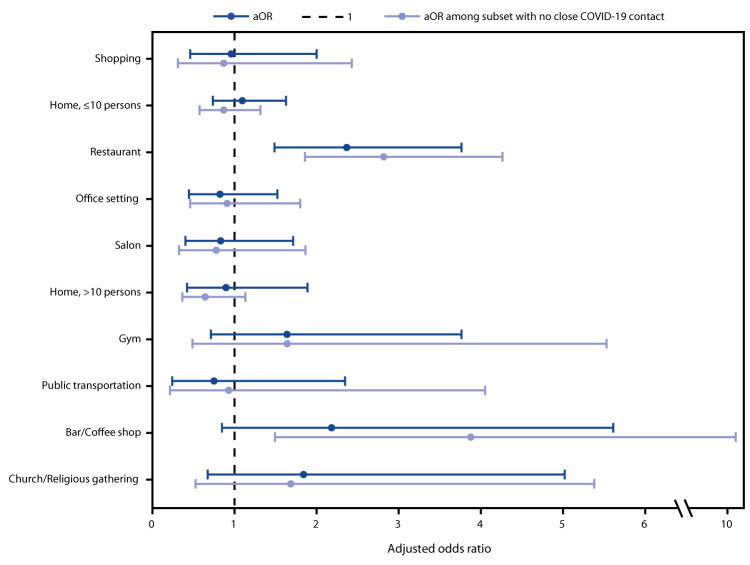
Adjusted odds ratio (aOR)* and 95% confidence intervals for community exposures^†^ associated with confirmed COVID-19 among symptomatic adults aged ≥18 years (N = 314) — United States, July 1–29, 2020 **Abbreviation:** COVID-19 = coronavirus disease 2019. * Adjusted for race/ethnicity, sex, age, and reporting at least one underlying chronic medical condition. Odds ratios were estimated using unconditional logistic regression with generalized estimating equations, which accounted for Influenza Vaccine Effectiveness in the Critically Ill Network site-level clustering. A second model was restricted to participants who did not report close contact to a person known to have COVID-19 (n = 225). ^†^ Community exposure questions asked were “In the 14 days before feeling ill about how often did you: shop for items (groceries, prescriptions, home goods, clothing, etc.); have people visit you inside your home or go inside someone else's home where there were more than 10 people; have people visit you inside your home or go inside someone else's home where there were 10 people or less; go to church or a religious gathering/place of worship; go to a restaurant (dine-in, any area designated by the restaurant including patio seating); go to a bar or coffee shop (indoors); use public transportation (bus, subway, streetcar, train, etc.); go to an office setting (other than for healthcare purposes); go to a gym or fitness center; go to a salon or barber (e.g., hair salon, nail salon, etc.).” Response options were coded as never versus at least once in the 14 days before illness onset.

## Discussion

In this investigation, participants with and without COVID-19 reported generally similar community exposures, with the exception of going to locations with on-site eating and drinking options. Adults with confirmed COVID-19 (case-patients) were approximately twice as likely as were control-participants to have reported dining at a restaurant in the 14 days before becoming ill. In addition to dining at a restaurant, case-patients were more likely to report going to a bar/coffee shop, but only when the analysis was restricted to participants without close contact with persons with known COVID-19 before illness onset. Reports of exposures in restaurants have been linked to air circulation (*7*). Direction, ventilation, and intensity of airflow might affect virus transmission, even if social distancing measures and mask use are implemented according to current guidance. Masks cannot be effectively worn while eating and drinking, whereas shopping and numerous other indoor activities do not preclude mask use.

Among adults with COVID-19, 42% reported close contact with a person with COVID-19, similar to what has been reported previously (*4*). Most close contact exposures were to family members, consistent with household transmission of SARS-CoV-2 (*8*). Fewer (14%) persons who received a negative SARS-CoV-2 test result reported close contact with a person with known COVID-19. To help slow the spread of SARS-CoV-2, precautions should be implemented to stay home once exposed to someone with COVID-19,** in addition to adhering to recommendations to wash hands often, wear masks, and social distance.^††^ If a family member or other close contact is ill, additional prevention measures can be taken to reduce transmission, such as cleaning and disinfecting the home, reducing shared meals and items, wearing gloves, and wearing masks, for those with and without known COVID-19.^§§^

The findings in this report are subject to at least five limitations. First, the sample included 314 symptomatic patients who actively sought testing during July 1–29, 2020 at 11 health care facilities. Symptomatic adults with negative SARS-CoV-2 test results might have been infected with other respiratory viruses and had similar exposures to persons with cases of such illnesses. Persons who did not respond, or refused to participate, could be systematically different from those who were interviewed for this investigation. Efforts to age- and sex-match participating case-patients and control-participants were not maintained because of participants not meeting the eligibility criteria, refusing to participate, or not responding, and this was accounted for in the analytic approach. Second, unmeasured confounding is possible, such that reported behaviors might represent factors, including concurrently participating in activities where possible exposures could have taken place, that were not included in the analysis or measured in the survey. Of note, the question assessing dining at a restaurant did not distinguish between indoor and outdoor options. In addition, the question about going to a bar or coffee shop did not distinguish between the venues or service delivery methods, which might represent different exposures. Third, adults in the study were from one of 11 participating health care facilities and might not be representative of the United States population. Fourth, participants were aware of their SARS-CoV-2 test results, which could have influenced their responses to questions about community exposures and close contacts. Finally, case or control status might be subject to misclassification because of imperfect sensitivity or specificity of PCR-based testing (*9*,*10*).

This investigation highlights differences in community and close contact exposures between adults who received a positive SARS-CoV-2 test result and those who received a negative SARS-CoV-2 test result. Continued assessment of various types of activities and exposures as communities, schools, and workplaces reopen is important. Exposures and activities where mask use and social distancing are difficult to maintain, including going to locations that offer on-site eating and drinking, might be important risk factors for SARS-CoV-2 infection. Implementing safe practices to reduce exposures to SARS-CoV-2 during on-site eating and drinking should be considered to protect customers, employees, and communities^¶¶^ and slow the spread of COVID-19.

**Summary**

**What is already known about the topic?**

Community and close contact exposures contribute to the spread of COVID-19.

**What is added by this report?**

Findings from a case-control investigation of symptomatic outpatients from 11 U.S. health care facilities found that close contact with persons with known COVID-19 or going to locations that offer on-site eating and drinking options were associated with COVID-19 positivity. Adults with positive SARS-CoV-2 test results were approximately twice as likely to have reported dining at a restaurant than were those with negative SARS-CoV-2 test results.

**What are the implications for public health practice?**

Eating and drinking on-site at locations that offer such options might be important risk factors associated with SARS-CoV-2 infection. Efforts to reduce possible exposures where mask use and social distancing are difficult to maintain, such as when eating and drinking, should be considered to protect customers, employees, and communities.

## Footnotes

* Baystate Medical Center, Springfield, Massachusetts; Beth Israel Deaconess Medical Center, Boston, Massachusetts; University of Colorado School of Medicine, Aurora, Colorado; Hennepin County Medical Center, Minneapolis, Minnesota; Intermountain Healthcare, Salt Lake City, Utah; Ohio State University Wexner Medical Center, Columbus, Ohio; Wake Forest University Baptist Medical Center, Winston-Salem, North Carolina; Vanderbilt University Medical Center, Nashville, Tennessee; John Hopkins Hospital, Baltimore, Maryland; Stanford University Medical Center, Palo Alto, California; University of Washington Medical Center, Seattle, Washington). Participating states include California, Colorado, Maryland, Massachusetts, Minnesota, North Carolina, Ohio, Tennessee, Utah, and Washington.

† Other languages included Spanish, Arabic, Vietnamese, Portuguese, and Russian.

§ Activity was determined to meet the requirements of public health surveillance as defined in 45 CFR 46.102(l)(2).

¶ Cardiac condition, hypertension, asthma, chronic obstructive pulmonary disease, immunodeficiency, psychiatric condition, diabetes, or obesity.

** https://www.cdc.gov/coronavirus/2019-ncov/if-you-are-sick/quarantine.html.

†† https://www.cdc.gov/coronavirus/2019-ncov/prevent-getting-sick/index.html.

§§ https://www.cdc.gov/coronavirus/2019-ncov/if-you-are-sick/index.html.

¶¶
https://www.cdc.gov/coronavirus/2019-ncov/daily-life-coping/personal-social-activities.html#restaurant ; https://www.cdc.gov/coronavirus/2019-ncov/community/organizations/business-employers/bars-restaurants.html ; https://www.cdc.gov/coronavirus/2019-ncov/images/community/Rest_Bars_RiskAssessment.jpg .
