# *Erratum:* Vol. 69, No. 36

**DOI:** 10.15585/mmwr.mm6938a7

**Published:** 2020-09-25

In the report “Community and Close Contact Exposures Associated with COVID-19 Among Symptomatic Adults ≥18 Years in 11 Outpatient Health Care Facilities — United States, July 2020,” on page 1262, the e-mail for contact information has been updated to eocevent101@cdc.gov.

